# Mortality Trends from 2003 to 2009 among Adolescents and Young Adults in Rural Western Kenya Using a Health and Demographic Surveillance System

**DOI:** 10.1371/journal.pone.0047017

**Published:** 2012-11-05

**Authors:** Penelope A. Phillips-Howard, Frank O. Odhiambo, Mary Hamel, Kubaje Adazu, Marta Ackers, Anne M. van Eijk, Vincent Orimba, Anja van’t Hoog, Caryl Beynon, John Vulule, Mark A. Bellis, Laurence Slutsker, Kevin deCock, Robert Breiman, Kayla F. Laserson

**Affiliations:** 1 Liverpool School of Tropical Medicine, Liverpool, United Kingdom; 2 KEMRI/CDC Research and Public Health Collaboration, Kisumu, Kenya; 3 Centers for Disease Control and Prevention, Atlanta, Georgia, United States of America; 4 Academic Medical Center, University of Amsterdam, Amsterdam, The Netherlands; 5 Centre for Public Health, Liverpool John Moores University, Liverpool, United Kingdom; 6 Centers for Disease Control and Prevention, Nairobi, Kenya; University of Massachusetts Medical School, United States of America

## Abstract

**Background:**

Targeted global efforts to improve survival of young adults need information on mortality trends; contributions from health and demographic surveillance system (HDSS) are required.

**Methods and Findings:**

This study aimed to explore changing trends in deaths among adolescents (15–19 years) and young adults (20–24 years), using census and verbal autopsy data in rural western Kenya using a HDSS. Mid-year population estimates were used to generate all-cause mortality rates per 100,000 population by age and gender, by communicable (CD) and non-communicable disease (NCD) causes. Linear trends from 2003 to 2009 were examined. In 2003, all-cause mortality rates of adolescents and young adults were 403 and 1,613 per 100,000 population, respectively, among females; and 217 and 716 per 100,000, respectively, among males. CD mortality rates among females and males 15–24 years were 500 and 191 per 100,000 (relative risk [RR] 2.6; 95% confidence intervals [CI] 1.7–4.0; p<0.001). NCD mortality rates in same aged females and males were similar (141 and 128 per 100,000, respectively; p = 0.76). By 2009, young adult female all-cause mortality rates fell 53% (χ^2^ for linear trend 30.4; p<0.001) and 61.5% among adolescent females (χ^2^ for linear trend 11.9; p<0.001). No significant CD mortality reductions occurred among males or for NCD mortality in either gender. By 2009, all-cause, CD, and NCD mortality rates were not significantly different between males and females, and among males, injuries equalled HIV as the top cause of death.

**Conclusions:**

This study found significant reductions in adolescent and young adult female mortality rates, evidencing the effects of targeted public health programmes, however, all-cause and CD mortality rates among females remain alarmingly high. These data underscore the need to strengthen programmes and target strategies to reach both males and females, and to promote NCD as well as CD initiatives to reduce the mortality burden amongst both gender.

## Introduction

While most global efforts to prevent mortality among young people focus on children below 5 years of age, there are significant health gains to be made among adolescent children and young adults, however, targeted efforts for this are hampered by a lack of data [1]–[3]. Of the estimated 2.6 million deaths occurring globally among adolescents and young adults (AYA) in 2004, two out of three deaths were in sub-Saharan Africa (SSA) and south-east Asia [1]. SSA has the highest AYA mortality rates for maternal, communicable disease, non-communicable disease, and injuries for both genders [1]. For example, among an estimated 10 million AYA aged 15–24 years living with HIV globally, 62% live in SSA [1], [3]–[6]. In 2004, disability adjusted life-years, comprising years of life disability as well as years of life lost, were 2.5 times higher in AYA aged 10–24 years in SSA compared with worldwide, and substantially higher than other low and middle income countries [3]. Further, in SSA, disability adjusted life-years comprise an equal proportion of years of life lost and years of life disability, whereas other regions mostly represent years of life disability [3]. Aggregated international data suggest mortality rates among AYA in SSA vary by age, but generally rates rise after early adolescence (10–14 years) and peak in young adulthood (20–24 years) [1]. Among young females, mortality is reportedly due to the burden of sexual and reproductive health threats, while injuries predominate among young males [1]–[4], [6].

Concern has been raised on the underinvestment in public health interventions for adolescents and young adults, and that investment for prevention against some threats, such as injuries, have fallen behind HIV/AIDS and reproductive health [3], [7], [8]. While a number of recent publications explored changing trends in all-cause and disease-specific mortality in adults in SSA [9]–[14], there is a dearth of literature on mortality ascertainment among AYA [15]. If global health targets are to include a stronger focus on the wider health concerns of AYA, longitudinal data would be helpful to define the needs and priorities, and how they evolve and are impacted by interventions. Quantification of various causes of death is imprecise in low and middle income countries, particularly in SSA, and sharing of available data for public health benefit is advocated [16]. Verbal autopsy, generated in subpopulations covered by health and demographic surveillance systems (HDSS) and standardised through the International Network for the Demographic Evaluation of Populations and Their Health in Developing Countries (INDEPTH), forms an important basis for mortality ascertainment in rural SSA [17]. INDEPTH is a global network of members who conduct longitudinal health and demographic evaluation of populations in low- and middle-income countries [18]. Data generated from the Kenya Medical Research Institute (KEMRI)/US Centers for Disease Control and Prevention (CDC) HDSS research site in rural western Kenya [19], [20], an INDEPTH Network member, provide local data to inform planning of programmes directed at health issues affecting the population. Data from the KEMRI/CDC HDSS have been utilised to examine the impact of TB disease on mortality [9], and HIV care and treatment service uptake on adult population-level mortality [14]. HDSS data were also examined to explore trends and characteristics of under-5 child mortality between 2003 and 2009 [21]. In this study, we aim to utilise HDSS data to explore the characteristics and trends in mortality, specifically among the highest risk AYA, aged 15–24 years, to identify changes in mortality in an area with expanding HIV treatment and care services, and discuss implications for public health programmes.

## Materials and Methods

### Study Site and Population

During the study period, the KEMRI/CDC HDSS study site was located in a rural part of Nyanza Province in western Kenya in Asembo (Rarieda District) and Gem (Yala and Wagai Divisions), Siaya District [19], [20], [22]. The area included 217 villages spread over a 500 km^2^ area along the shores of Lake Victoria, with a mid-year population of 136,448 in 2003 rising to 146,081 by 2009. Among AYA aged 15–24 years, the gender breakdown is relatively equal, with a mean (annually fluctuating) mid-year annual population of 14,780 males and 14,502 females (total 29,282). The population, mainly subsistence farmers, are almost exclusively members of the Luo ethnic group and have been described in detail elsewhere [19], [22], [23]. Inhabitants live in family compounds comprising one or more (average 2.1) houses surrounded by their land. The society is polygynous with approximately a third of males having more than one wife [23]. The population is impoverished with a mean ‘wealth index’ previously estimated to be $600 to $700 per compound [24]. HIV [5], [25]–[27], TB [9], [28]–[30], malaria [31]–[34], schistosomiasis [35]–[37], and suboptimal water quality, sanitation and hygiene [38]–[41], are leading causes of morbidity and mortality within the study area.

### Health and Demographic Surveillance System (HDSS)

The population was registered and households were geo-spatially located during an insecticide treated bednet trial [22]. The HDSS site was registered in 2001 as a member of the INDEPTH Network [19], [20]. A household census is conducted throughout the study area tri-annually to capture births, pregnancies, deaths, in- and out- migration, and economic data. These data provide mid-year population denominators, stratified by age group and gender.

### Verbal Autopsy (VA)

All resident deaths reported to field staff during census are followed up with a visit to households to validate deaths and record events surrounding death, using a standardized verbal autopsy (VA) questionnaire. Residents are defined as all persons residing in the study site for 4 months or more, precluding transient residents and visitors. VA is conducted using standardised WHO questionnaires endorsed by INDEPTH, for all deaths occurring in the HDSS [18], [20]. For this analysis, we utilized the adult questionnaire (15 years and above). A previous one year review of deaths examined data from 2003 and describes the VA methodology in detail [42]. Resident identification numbers allow linkage of each death with HDSS data. Parents or spouses are identified as the first respondents. VA interviews are performed, at least one month (average 3 months) after the death to respect the mourning period, while still facilitating recall. Absence of an adult in the home is recorded as a non-VA interview, enabling only verification that death occurred and collection of minimal demographic indices. VA forms are reviewed independently by at least two clinical officers and cause of death assigned. In 2006, “Sample Vital Registration with Verbal Autopsy (SAVVY)” was adopted at the KEMRI/CDC HDSS (and across INDEPTH sites) to strengthen vital event monitoring and measurement. SAVVY constitutes a resource library of best practice to improve the quality of civil registration, harmonized to the WHO International Classification of Disease [43]. This facilitated attribution of the cause of death.

### Ethical Considerations

Following cultural customs, compound heads provide written consent for all compound members to participate in the HDSS activities. Any individual can refuse to participate at any time. The HDSS protocol and consent procedures, including surveillance and VA activities, were approved by KEMRI (#1801) and CDC Institutional Review Boards (#3308). All HDSS census and VA data are maintained on a secure server with access only by authorized researchers. Named data are securely stored in a MS-SQL database and only authorized data personnel have access rights. Datasets used by scientists for analysis are stripped of names to protect identity.

**Table 1:** Characteristics of deaths a in late adolescents and young adults aged 15 to 24 years, documented through verbal autopsy.

Variable	N^b^	Value	Female (%)	Male (%)	Total (%)	RR^fem^ (95% CI)	χ^2^	p value
Age	967	15–19y	161 (24.7)	132 (41.8)	293 (30.3)	0.59 (0.49–0.71)	29.25	<0.001
		20–24y	490 (75.3)	184 (58.2)	674 (69.7)			
Area	967	Asembo	272 (41.8)	125 (39.6)	397 (41.1)	1.06 (0.90–1.24)	0.44	0.509
		Gem	379 (58.2)	190 (60.4)	570 (58.9)			
SES^c^	909	MCA^c^1–2	241 (39.3)	93 (31.4)	334 (36.7)	1.25 (1.03–1.52)	5.35	0.021
		MCA^c^3–5	372 (60.7)	203 (68.6)	575 (63.6)			
Ever Married	814	Yes	330 (60.8)	61 (22.5)	391 (48.0)	2.70 (2.14–3.40)	106.4	<0.001
		No	213 (39.2)	210 (77.5)	423 (52.0)			
Divorced^d^	346	Yes	74 (25.8)	19 (32.2)	93 (26.9)	0.80 (0.53–1.22)	1.03	0.311
		No	213 (74.2)	40 (67.8)	253 (73.1)			
Widowed	391	Yes	43 (13.0)	2 (3.3)	45 (11.5)	3.97 (0.99–16.0)	4.81	0.028
		No	287 (87.0)	59 (96.7)	346 (88.5)			
Primary School	642	Attend yes	381 (90.9)	203 (91.0)	584 (91.0)	0.99 (0.95–1.05)	0.00	0.966
		Attend no	38 (9.1)	20 (9.0)	58 (9.0)			
Secondary School	788	Attend yes	65 (12.4)	38 (14.3)	103 (13.1)	0.87 (0.60–1.26)	0.57	0.452
		Attend no	458 (87.6)	227 (85.7)	685 (86.9)			
Place Died	818	Home	406 (74.4)	189 (69.5)	595 (72.7)	1.07 (0.98–1.17)	2.18	0.140
		+HHF^e^	140 (25.6)	83 (30.5)	223 (27.3)			

NOTE. RR^fem^, Relative risk for females compared with males; CI, confidence interval; χ^2^, chi-squared.

a All-cause mortality, includes persons who died with no cause of death allocated (undetermined).

b Numbers vary due to missing data (for example, a relative’s non-response), or within categories such as divorced or widowed among those reportedly ever married.

c Socio-economic status (SES) defined through Multiple Correspondence Analysis (MCA) wealth quintiles 1 = poorest 5 = least poor, ranking then collapsed into 1–2 (most poor), 3–5 (less poor).

d Divorced or separated from partner at time of death, denominator restricted to those reportedly ever married.

e +HHF represents place of death in hospital, health facility, or to/from HHF.

### Analyses

Data were extracted from the HDSS database for all deaths occurring among residents aged between 15 to 24 years of age at the time of death, between January 2003 and December 2009. Data transformation and analyses were conducted using SPSS for Windows (Release v18.0), and EpiInfo Stat Calc (CDC Atlanta, USA). Analyses on proportions and rates per 100,000 population were conducted on all-cause; and grouped into communicable disease (CD), and non-communicable disease (NCD) causes. The category of NCD included injuries, maternal (including septicaemia), cancers and nutritional causes.

Mean age of death among all AYA aged 15–24 years is presented with standard deviation (SD), for all-cause mortality by gender, and for key diseases. Analyses are stratified into adolescence (15–19 years old) and young adulthood (20–24 years old). Mortality rates per 100,000 were estimated by year and age category, using mid-year population-point estimates generated from the HDSS census. Dates of death were grouped per year to facilitate calculation of annual mortality rates per age category and by gender. Key social and demographic characteristics generated through the HDSS for analyses here included marital status (ever married; divorced or widowed at time of death), place of death (home or health facility; comprising clinic, hospital, on route to/from health facility); education (attended and completed primary school), and socio-economic status (SES). Routinely collected SES indicators such as occupation of household head, primary source of drinking water, use of cooking fuel, in-house assets (e.g., lantern lamp, sofa, bicycle, radio, TV) and livestock (poultry, pigs, donkey cattle, sheep, goats) [24], were used to calculate an SES index as a weighted average using multiple correspondence analysis [44]. This ranked households into SES quintiles with the first quintile representing the poorest and the fifth representing the least poor; for some analyses this was collapsed into most (1–2) and less (3–5) poor.

**Figure 1:**
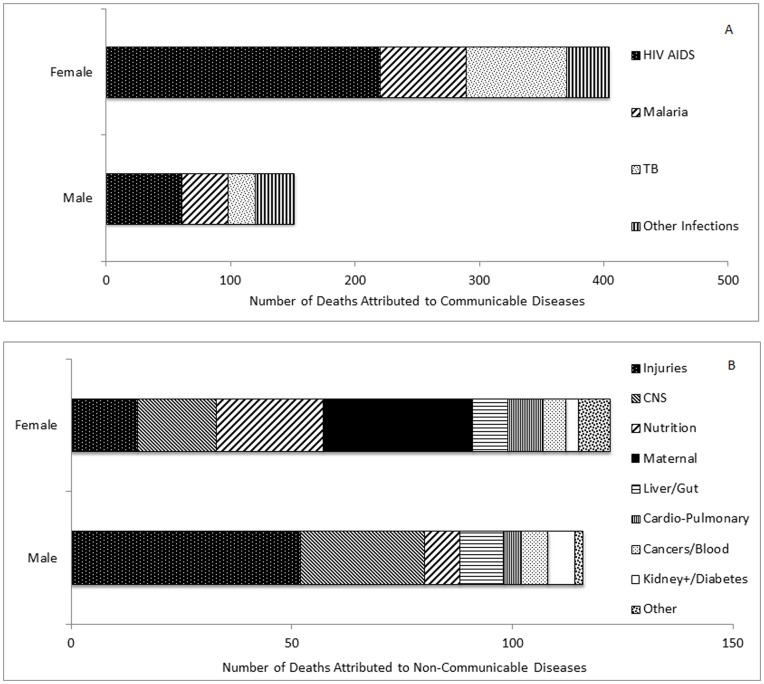
Number of deaths by primary cause in adolescents and young adults, 2003–2009, by primary cause and gender. A, Communicable diseases; B, Non-communicable diseases. Note axis change between Figure 1A and Figure1B.

Analyses to examine trends in mortality rates per 100,000 population over time were conducted for all-cause, CD and NCD sub-strata. Chi-squared (χ^2^) test for linear trend determined the statistical significance of changing rates by gender over time (2003 to 2009). Differences between groups were determined using Pearson’s χ^2^ test, and the level of significance was set at 5% or less. Mantel-Haenszel Relative Risks (RR), with Taylor Series 95% confidence intervals (CI), was used to compare mortality rates between genders, by year of death. Unless stated, RR compares female to male rates; where rates are significantly higher for males, reciprocal values are given (RR^male^).We stratified RR analyses for mortality rates into the two age groups, by gender, by year for all-cause, CD, and NCD mortality, generating a summary χ^2^, with a MH weighted RR and Greenlands-Robins 95% CI.

## Results

### Socio-demographics

Of 12,041 documented deaths occurring during the study period in adults aged 15 years and above in Asembo and Gem, 967 (8.0%) were among AYA (n = 293, 30.3% 15–19 years; n = 674, 69.7% 20–24 years, table 1). Females constituted two-thirds (651; 67.3%) of deaths. Young adult females (20–24 years) were disproportionately represented, comprising three quarters (75.3%) of all female deaths. The mean age of death of all AYA deaths was 21.3 years (standard deviation; SD 2.7 years). Just over a third of deaths were in the two lowest SES quintiles, with a higher proportion of females (39.3%) than males (31.4%) represented (relative risk [RR] 1.25; 95% confidence intervals [CI] 1.0–1.5, table 1); this compares with 28% and 25% in females and males, aged 18–24 years within the population (data not shown). Significantly more females (60.8%) had been ever married compared with males (22.5%; RR 2.7, 95% CI 2.1–3.4). However, among those AYA who had ever been married, 26.9% were divorced or separated at the time of their death, with no significant difference by gender. This compares with a divorce rate of <5% among those 18–24 years in the population (data not shown). Among ever married, a further 11.5% were widowed at the time of their death; here there was a strong association with gender, with 13.0% of females compared with just 3.3% of males reported to have been widowed (RR 3.97; 95% CI 1.0–16.0). Widowhood among the general population in this age group is rare, with rates recorded of 2% and 1% among females and males aged 18–24 years (data not shown). Of 642 with reported primary school status, 91% of deaths in both gender had attended school, however, less than half (46.6%; 49.6% of females and 40.8% of males, p = 0.033) completed their primary school education. Only 13.1% attended secondary school, with no significant difference by gender (table 1). The majority (72.7%) of AYA died at home, among both males (69.5%) and females (74.4%).

**Table 2:** Main causes of death^a^ among adolescents and young adults (15–24 years), by gender.

Cause		Female (%) n = 526	Male (%) n = 267	Total (%) n = 793	RR^fem^ (95% CI)	χ^2^	p value
CD^b^	Yes	404 (76.8)	151 (56.6)	555 (70.0)	1.36 (1.21–1.52)	34.6	<0.001
	No	122 (23.2)	116 (43.4)	238 (30.0)			
HIV/TB^c^	Yes	301 (57.2)	83 (31.1)	384 (48.4)	1.84 (1.52–2.23)	48.5	<0.001
	No	225 (42.8)	184 (68.9)	409 (51.6)			
HIV	Yes	220 (41.8)	61 (22.8)	281 (35.4)	1.83 (1.44–2.33)	27.9	<0.001
	No	306 (58.2)	206 (77.2)	512 (64.6)			
TB	Yes	81 (15.4)	22 (8.2)	103 (13.0)	1.87 (1.20–2.92)	8.03	0.005
	No	445 (84.6)	245 (91.8)	690 (87.0)			
Malaria	Yes	69 (13.1)	37 (14.3)	106 (13.4)	0.95 (0.65–1.37)	0.08	0.77
	No	457 (86.9)	230 (86.1)	687 (86.6)			
Oth.Infections	Yes	34 (6.5)	31 (11.6)	65 (8.2)	0.56 (0.35–0.89)	6.23	0.013
	No	492 (93.5)	236 (88.4)	728 (91.8)	[1.80(1.13–2.86)]^d^		
Injuries	Yes	15 (2.9)	52 (19.5)	67 (8.4)	0.15 (0.08–0.26)	63.3	<0.001
	No	511 (97.1)	215 (80.5)	726 (91.6)	[6.85 (3.92–11.9)]		
CNS	Yes	18 (3.4)	28 (10.5)	46 (5.8)	0.33 (0.18–0.58)	16.2	<0.001
	No	508 (96.6)	239 (89.5)	747 (94.2)	[3.06 (1.73–5.43)]		
Nutritional	Yes	24 (4.6)	8 (3.0)	32 (4.0)	1.52 (0.69–3.34)	1.12	0.289
	No	502 (95.4)	259 (97.0)	761 (96.0)			
Maternal	Yes	34 (6.5)	–––	34 (4.3)	–	18.0	<0.001
	No	492 (93.5)	267 (100 )	759 (95.7)			
Respiratory	Yes	4 (0.8)	1 (0.4)	5 (0.6)	2.03 (0.23–18.08)	0.42	0.516
	No	522 (99.2)	266 (99.6)	788 (99.4)			
Gut	Yes	4 (0.8)	4 (1.5)	8 (1.0)	0.51 (0.13–2.01)	1.97	0.326
	No	522 (99.2)	263 (98.5)	785 (99.0)			
Liver	Yes	4 (0.8)	6 (2.2)	10 (1.3)	0.34 (0.10–1.19)	3.14	0.076
	No	522 (99.2)	261 (97.8)	783 (98.7)			
Cancer	Yes	4 (0.8)	3 (1.1)	7 (0.9)	0.68 (0.15–3.02)	0.27	0.605
	No	522 (99.2)	264 (98.9)	786 (99.1)			
Cardio-vascular	Yes	4 (0.8)	3 (1.1)	7 (0.9)	0.68 (0.15–3.02)	0.27	0.605
	No	522(99.2)	264 (98.9)	786 (99.1)			
Renal/Urinary	Yes	2 (0.4)	4 (1.5)	6 (0.8)	0.25 (0.05–1.38)	2.93	0.086
	No	524 (99.6)	263 (98.5)	787 (99.2)			
Diabetes	Yes	1 (0.2)	2 (0.7)	3 (0.4)	0.25 (0.02–2.79)	1.47	0.226
	No	525 (99.8)	265 (99.3)	790 (99.6)			

NOTE. RR^fem^, Relative risk for females compared with males; CI, confidence interval; χ^2^, chi-squared.

a Statistics presented exclude deaths with undetermined cause (n = 174); of 238 NCD deaths, 13 ‘other’ NCDs are excluded from main cause of death analysis.

b CD, communicable diseases (HIV, TB, malaria, other common infections).

c HIV/TB is the combination of all deaths diagnosed with either TB or HIV as the cause of death.

d Significantly higher proportion of deaths in males, inverse RR^males^ presented [in brackets].

### Causes of Death

Ascribed cause of death was available for 793 (82.1%) of 967 deaths. Among the 174 deaths with no cause determined, age, gender, SES and place of death were comparable with deaths attributed to a cause. Among those with a cause, 70.0% fell within the communicable disease classification, and 30.0% were classified with a non-communicable disease (figure 1).

**Table 3:** Trends in all-cause mortality rates per 100,000 by gender and age group.

		Female MR^a^	Male MR^a^	RR^fem^ (95% CI)	χ^2^	P value	M:F Ratio
15–24y	2003	874	390	2.24 (1.63–3.08)	26.4	<0.001	0.45
	2004	796	307	2.59 (1.83–3.67)	31.2	<0.001	0.39
	2005	661	281	2.35 (1.63–3.39)	22.2	<0.001	0.43
	2006	642	310	2.07 (1.45–2.95)	17.0	<0.001	0.48
	2007	656	224	2.93 (1.98–4.32)	32.0	<0.001	0.34
	2008	466	298	1.56 (1.08–2.27)	5.70	0.017	0.64
	2009	414	334	1.24 (0.85–1.80)	1.28	0.26	0.81
	χ^2^ LT^b^	34.14	0.99	MHRR^c^ 2.08 (1.82–2.38); Summary χ^2^ 121.55; p<0.001			
	P value	<0.001	0.33				
15–19y	2003	403	217	1.86 (1.08–3.22)	5.1	0.02	0.54
	2004	323	238	1.36 (0.78–2.37)	1.2	0.28	0.74
	2005	292	138	2.11 (1.08–4.13)	5.0	0.25	0.47
	2006	255	217	1.18 (0.64–2.17)	0.3	0.60	0.85
	2007	262	194	1.35 (0.73–2.52)	0.9	0.34	0.74
	2008	197	180	1.09 (0.56–2.14)	0.1	0.79	0.91
	2009	155	240	0.65 (0.33–1.28)	1.6	0.21	1.55
	χ^2^ LT^b^	11.94	0.001	MHRR^c^ 1.33 (1.06–1.67); Summary χ^2^ 5.65; p = 0.02			
	P value	<0.001	0.97				
20–24y	2003	1613	716	2.25 (1.53–3.32)	17.7	<0.001	0.44
	2004	1540	433	3.56 (2.23–5.68)	32.5	<0.001	0.28
	2005	1217	541	2.25 (1.45–3.49)	14.0	<0.001	0.44
	2006	1166	465	2.51 (1.60–3.92)	17.4	<0.001	0.40
	2007	1186	273	4.34 (2.53–7.45)	34.1	<0.001	0.23
	2008	817	484	1.69 (1.08–2.65)	5.3	0.02	0.59
	2009	754	482	1.57 (0.98–2.49)	3.6	0.06	0.64
	χ^2^ LT^b^	30.40	2.73	MHRR^c^ 2.43 (2.05–2.88); Summary χ^2^ 112.8; p<0.001			
	P value	<0.001	0.10				

NOTE. RR^fem^, Relative risk for females compared with males; CI, confidence interval; χ^2^, chi-squared.

a MR, mortality rates per 100,000, note all-cause mortality includes deaths with undetermined cause, and are thus higher than combined communicable and non-communicable disease mortality rates.

b χ^2^ LT, chi-squared for linear trend.

c MHRR, Mantel Haenszel weighted relative risk, and Greenlands-Robins 95% confidence intervals.

#### Communicable diseases (CD)

CD included HIV, TB, malaria and other infections (figure 1a). Three quarters (76.8%) of deaths among females, compared with a half (56.6%) of male deaths were from a CD (table 2). This largely reflects the burden of HIV, identified as the primary cause in 281 (35.4%) of all deaths, and 50.6% of CD deaths. HIV was attributed to 41.8% of all female and 22.8% of all male deaths, and constituted 54.5% and 40.4% of female and male CD deaths, respectively. AYA females, thus, had close to a two-fold (RR 1.8, 95% CI 1.4–2.3) higher proportion of deaths attributed to HIV compared with males. Malaria was reported as the second overall leading cause of death, in 13.4% of all deaths, constituting 19.1% of CD deaths, with no gender difference. TB was categorized as the direct cause in 13.0% of all deaths, constituting 18.6% of all CD deaths, with a higher proportion among females compared with males (15.4% versus 8.2%; p = 0.005). HIV and TB combined constituted 69.2% of all CD deaths and 48.0% of all attributed AYA deaths; contributing 57.2% of all female and 31.1% of all male diagnosed deaths (RR 1.8, 95% CI 1.5–2.2). Other infections contributed to 11.7% of CD deaths and to 8.2% of all ascribed causes of death. More male (11.6%) than female (6.5%) deaths were from ‘other infections’ (RR^male^ 1.8, 95% CI 1.1–2.9). Among “other infections”, most common were meningitis (40%), pneumonia (29.2%), gastro-enteritis/dysentery/cholera (18.5%), typhoid and paratyphoid (6.2%), sepsis (4.6%), and rheumatic heart disease (1.5%).

**Figure 2:**
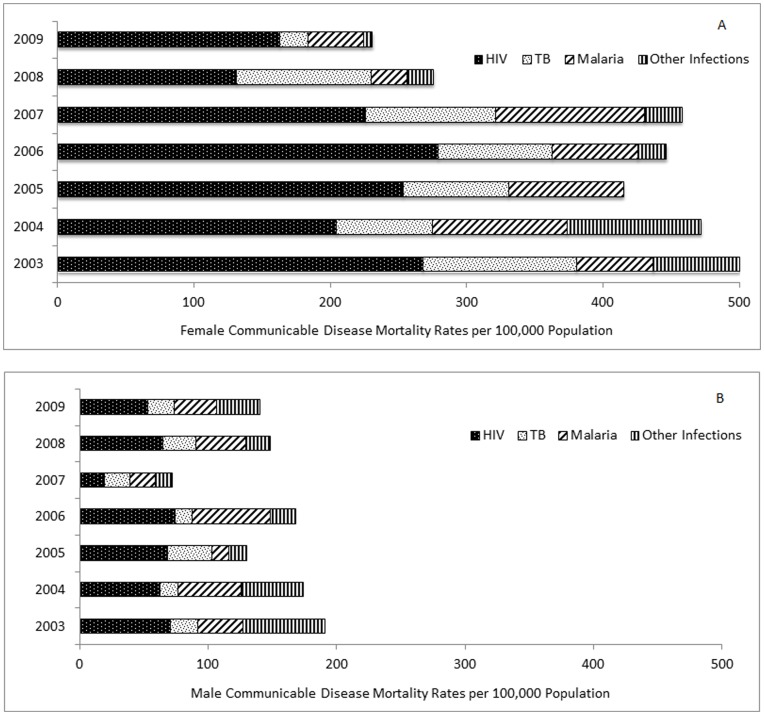
Communicable disease mortality rates in adolescents and young adults, 2003–2009, by primary cause. A, Female; B, Male.

**Table 4:** Trends in communicable disease mortality rates per 100,000 by gender and age group.

		Female MR^a^	Male MR^a^	RR^fem^ (95% CI)	χ^2^	P value	M:F Ratio
15–24y	2003	500	191	2.61(1.68–4.07)	19.6	<0.001	0.38
	2004	472	174	2.71 (1.71–4.28)	19.7	<0.001	0.37
	2005	415	130	3.18 (1.90–5.33)	21.6	<0.001	0.31
	2006	447	169	2.65 (1.67–4.20)	18.5	<0.001	0.38
	2007	458	73	6.31 (3.34–11.9)	42.3	<0.001	0.16
	2008	276	149	1.85 (1.11–3.08)	5.8	0.016	0.54
	2009	231	140	1.65 (0.96–2.83)	3.3	0.07	0.61
	χ^2^ LT^b^	17.80	2.40	MHRR^c^ 2.73 (2.26–3.28); Summary χ^2^ 119.28; p<0.001			
	P value	<0.001	0.12				
15–19y	2003	230	119	1.93 (0.93–4.03)	3.2	0.07	0.52
	2004	150	130	1.16 (0.53–2.53)	0.1	0.72	0.87
	2005	164	43	3.85 (1.27–11.8)	6.6	0.01	0.26
	2006	133	108	1.23 (0.52–2.90)	0.2	0.63	0.81
	2007	167	43	3.88 (1.28–11.8)	6.7	0.01	0.26
	2008	69	63	1.09 (0.35–3.39)	0.02	0.88	0.91
	2009	72	66	1.09 (0.35–3.39)	0.02	0.88	0.92
	χ^2^ LT^b^	8.75	3.48	MHRR^c^ 1.73 (1.22–2.43); Summary χ^2^ 9.39; p = 0.002			
	P value	0.003	0.06				
20–24y	2003	924	327	3.07 (1.73–5.45)	16.4	<0.001	0.35
	2004	979	256	3.82 (2.09–7.00)	22.0	<0.001	0.26
	2005	794	290	2.74 (1.53–4.91)	12.5	<0.001	0.37
	2006	870	268	3.24 (1.83–5.75)	18.3	<0.001	0.31
	2007	849	119	7.11 (3.24–15.6)	32.6	<0.001	0.14
	2008	545	284	1.92 (1.08–3.41)	5.1	0.02	0.52
	2009	440	258	1.64 (0.88–3.09)	2.4	0.12	0.59
	χ^2^ LT^b^	14.96	0.71	MHRR^c^ 2.99 (2.38–3.74); Summary χ^2^ 98.49; p<0.001			
	P value	<0.001	0.40				

NOTE. RR^fem^, Relative risk for females compared with males; CI, confidence interval; χ^2^, chi-squared.

a MR, mortality rates per 100,000; includes deaths ascribed to HIV, TB, malaria, and other infections (meningitis, pneumonia, gastro-intestinal, typhoid/paratyphoid, sepsis, and rheumatic heart disease).

b χ^2^ LT, chi-squared for linear trend.

c MHRR, Mantel Haenszel weighted relative risk, and Greenlands-Robins 95% confidence intervals.

#### Non-communicable diseases (NCD)

Deaths attributed to injuries (8.4% of all diagnosed causes) were predominantly among males (figure 1b), with 52 of 67 injuries (77.6%; RR^male^ 6.8; 95% CI 3.9–11.9; table 2), and ranked as the overall second highest cause among males after HIV (19.5% of all male deaths, and 44.8% of all male NCD deaths). Injuries were ascribed to poisonings/other trauma (47.8%), accidents (25.4%), assault (16.4%), and suicides (10.4%). Six of the seven recorded suicides were male. For females, deaths directly attributed to pregnancy and childbirth contributed a quarter (24.6%) of their NCD burden, with 6.5% of all female deaths overall ascribed maternal death as the prime cause. Reported causes were puerperal sepsis (29.4%), ante- or post-partum haemorrhage (17.6%), eclampsia (14.7%), abortion/miscarriage (11.8%), obstructed labour (8.8%), and ‘other complications’ (17.6%). Mortality attributed to a central nervous system (CNS) cause was predominantly (93.5%) attributed to seizures/epilepsy. Of the remaining NCD deaths, other disorders associated with hepatic (hepatic unspecified), respiratory (asthma), renal (nephrotic syndrome, renal failure), gut (obstruction, symptoms of abdomen/pelvis), or cardio-vascular (congestive cardiac failure), disorders each contributed <5% of the deaths among AYA (table 2).

**Figure 3:**
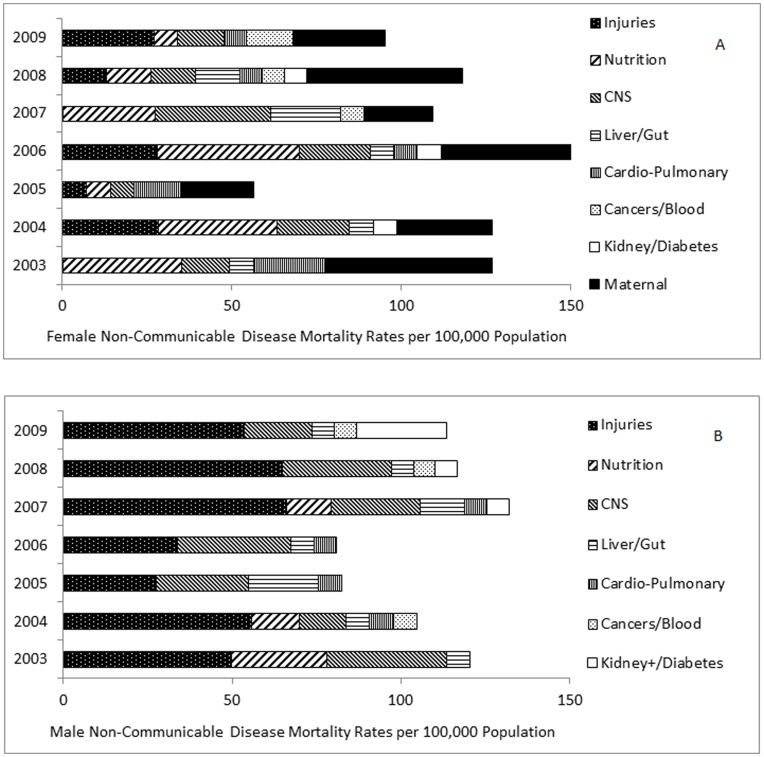
Non-communicable disease mortality rates in adolescents and young adults, 2003–2009, by primary cause. A, Female; B, Male. Causes of death aggregated.

**Figure 4:**
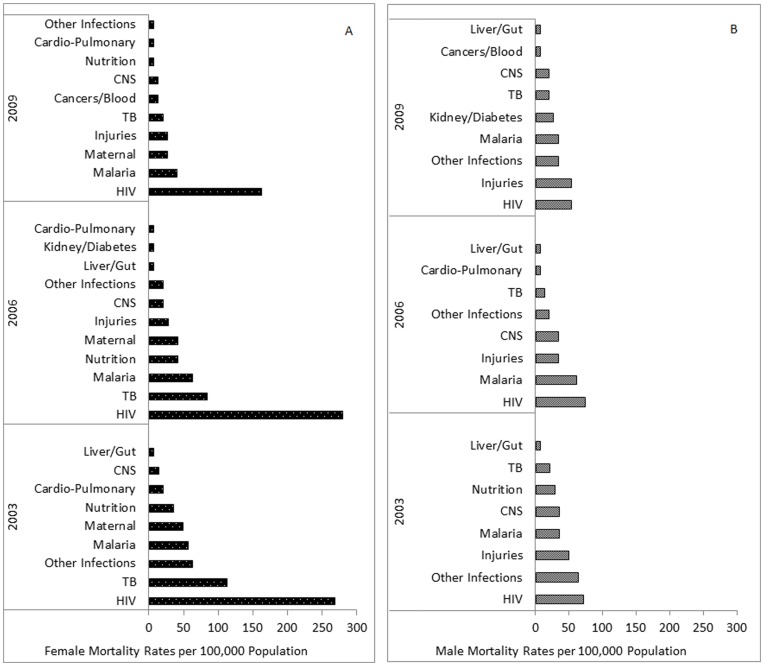
Mortality rates by top primary causes in 2003, 2006 and 2009. A, Female; B, Male. Causes of death aggregated.

**Table 5:** Trends in non-communicable disease mortality rates per 100,000 by gender and age group.

		Female MR^a^	Male MR^a^	RR^fem^ (95% CI)	χ^2^	P value	M:F Ratio
15–24y	2003	141	128	1.10 (0.58–2.09)	0.09	0.76	0.91
	2004	169	105	1.62 (0.85–3.08)	2.17	0.14	0.62
	2005	56	82	0.68 (0.28–1.66)	0.73	0.39	1.46
	2006	153	81	1.90 (0.94–3.83)	2.28	0.13	0.53
	2007	109	132	0.83 (0.43–1.60)	0.31	0.58	1.21
	2008	118	117	1.01 (0.53–1.95)	0.00	0.97	0.99
	2009	95	140	0.68 (0.34–1.33)	1.29	0.26	1.47
	χ^2^ LT^b^	1.49	0.63	MHRR^c^ 1.07 (0.83–1.38); Summary χ^2^ 0.21; p = 0.65			
	P value	0.22	0.43				
15–19y	2003	58	76	0.76 (0.24–2.39)	0.2	0.64	1.31
	2004	104	76	1.37 (0.51–3.68)	0.4	0.53	0.73
	2005	47	74	0.63 (0.18–2.14)	0.6	0.45	1.57
	2006	97	54	1.79 (0.59–5.48)	1.1	0.45	0.56
	2007	60	118	0.50 (0.18–1.45)	1.7	0.19	1.97
	2008	69	95	0.73 (0.26–2.05)	0.4	0.55	1.38
	2009	72	131	0.55 (0.21–1.46)	1.5	0.22	1.82
	χ^2^ LT^b^	0.018	2.28	MHRR^c^ 0.81 (0.55–1.20); Summary χ^2^ 0.9; 0.34			
	P value	0.93	0.13				
20–24y	2003	272	225	1.21 (0.56–2.63)	0.2	0.64	0.83
	2004	272	157	1.73 (0.73–4.07)	1.6	0.21	0.58
	2005	71	97	0.73 (0.20–2.72)	0.2	0.75	1.37
	2006	230	125	1.84 (0.74–4.55)	1.8	0.18	0.54
	2007	176	154	1.15 (0.48–2.77)	0.1	0.76	0.88
	2008	182	150	1.21 (0.51–2.87)	0.2	0.67	0.82
	2009	126	155	0.81(0.31–2.10)	0.2	0.67	1.23
	χ^2^ LT^b^	1.83	0.11	MHRR^c^ 1.25 (0.89–1.74); Summary χ^2^ 1.3; p = 0.239			
	P value	0.18	0.74				

NOTE. RR^fem^, Relative risk for females compared with males; CI, confidence interval; χ^2^, chi-squared.

a MR, mortality rates per 100,000, includes deaths ascribed to injuries, CNS, nutritional, maternal, respiratory, gut, liver, cancers, cardio-vascular, renal/urinary, and diabetes.

b χ^2^ LT, chi-squared for linear trend.

c MHRR, Mantel Haenszel weighted relative risk, and Greenlands-Robins 95% confidence intervals.

### Changing Patterns of Mortality

#### All-cause mortality rates

Between 2003 and 2009, the absolute number of deaths recorded annually decreased from 179 in 2003 to 111 in 2009. While the number of female deaths halved from 124 to 61, male deaths fell only marginally (from 55 to 50), resulting in the overall ratio of male to female deaths changing from 0.44 (55∶124) in 2003 to 0.82 (50∶61) by 2009. There was a 2-fold higher all-cause mortality rate among 15–24 year old females compared with same aged males in 2003 (RR 2.2; 1.6–3.1; table 3). Rates were significantly higher among females in both age groups. Annual all-cause mortality rates fell significantly from 2003 to 2009 among females but not males (table 3). Rates halved from 874 to 414 per 100,000 population for all AYA females, showing a significant linear trend, while a 14% reduction among males was not significant. Mortality reduction was significant among young adult females, reducing 53% from 1,613 to 754 per 100,000. In same aged males, deaths fell 33% from 716 to 482 but with no significant linear trend. By 2009, all-cause mortality rates were no longer significantly different by gender. Among adolescents, while all-cause mortality rates fell significantly among females by 61.5%, from 403 to 155 per 100,000, mortality rates were static for same aged males. This resulted in the rate of mortality among adolescent females dipping below that of males in 2009 (155 and 240 per 100,000, respectively), with no significant difference in mortality rates by gender in that year (table 3).

#### Communicable disease mortality rates

In 2003, CD mortality rates were over two and a half times higher among female AYA compared with males (500 versus 191 per 100,000; RR 2.6; 1.7–4.1; table 4; figure 2). By 2009 there had been a two-fold reduction among females, with rates halving significantly from 500 to 224 per 100,000 (figure 2a). This fall was not mirrored among males with rates decreasing non-significantly by 27% from 191 to 140 per 100,000 (figure 2b). In adolescents, CD mortality rates fell for both genders (69% females, 44.5% for males); the drop was statistically significant for females (χ^2^ for linear trend 8.75; p = 0.003), but not for males. Among young adults, CD mortality rates halved among females from 924 to 440 per 100,000 (χ^2^ for linear trend 15.0; p<0.001), but for same aged males, the fall of 21% from 327 to 258 per 100,000 was not associated with a significant linear trend. The fall in rates among females, together with marginal changes among males, resulted in no significant differences in CD mortality rates between females and males, overall, and by each age group by 2009 (table 4), although rates for females still exceeded those of males. The ratio of male to female CD mortality rates changed from 0.38 in 2003, to 0.61 by 2009.

#### Non-communicable disease mortality rates

Two main causes of death for NCD were injuries in males and maternal causes among females. NCD mortality rates in males and females showed no significant trends by year or by gender (figure 3). Including maternal mortality, the NCD-attributed mortality rate for AYA females aged 15–24 years ranged between 56 and 169 per 100,000 (table 5; figure 3a). There was no evidence of linear change in mortality rates over time. While NCD mortality rates for young adult females fell 53.7% from 272 to 126 per 100,000 between 2003 and 2009, the linear trend was not significant. Similar rates were recorded for males, with a comparable range as for females in both age groups (table 5), with no significant difference in rates by gender. Rates among males fluctuated over time with no significant trend in overall rates (figure 3b). By 2009, mortality rates associated with injury equalled HIV as the top cause of death among males (figure 4), and rose among females to constitute the 3^rd^ leading cause of death (figures 4a, 4b). The ratio of male to female NCD mortality rates changed from 0.91 in 2003, to 1.47 by 2009.

## Discussion

Despite calls to strengthen public health measures for adolescents and young adults, particularly for sub-Saharan Africa (SSA), publications examining mortality rates in persons over 15 years have aggregated adult age, preventing evaluation of the specific changes occurring among adolescents and young adults. Data presented here were generated from a longitudinal, consistently maintained, health and demographic surveillance system in a rural area of western Kenya, facilitating comparison by age and gender over time, broken down into communicable and non-communicable disease causes. We found significant reductions in all-cause and communicable disease mortality among all female AYA (aged 15–24 years) between 2003 and 2009; however, no reductions were found among same aged males, or for non-communicable diseases in either gender.

All-cause mortality rates halved among female AYA aged 15–24 years between 2003 and 2009. The very high burden of mortality among this population is demonstrated by comparison with the average all-cause mortality rates estimated for young women in SSA in global burden of disease (GBD) studies [1]. The average all-cause mortality rate for females aged 20–24 years in SSA was 522 per 100,000 in the GBD study for 2004, three-fold lower than the 1,540 per 100,000 calculated for same aged females in western Kenya in the same year (table 3). Even by 2009, when rates had halved to 754 per 100,000 in our western Kenya site, rates were still higher than the GBD 2004 estimated average [1].

In our analyses, three-quarters of female deaths were ascribed to communicable diseases, predominantly HIV. The prevalence of HIV among adults in Kenya was estimated to be around 7% in 2007. Nyanza Province (which includes our study site), bears the highest burden at 14.9% [45]. HIV sero-prevalence in females was found to rise rapidly during adolescence; in the HDSS study area the prevalence among 13–14 year olds was 1.3%, rising to 12.8% by 17 years of age [5], and was 10-fold higher in young females compared with same aged males [5]. Exposure of young females to HIV from older male partners [46], results in a high prevalence of HIV infected female widows [47]. In the study area female but not male widowhood was strongly associated with HIV [5]. In our study, 13% of AYA females who died were widowed compared with 3.3% of males. HIV-related deaths in SSA had peaked in 2004, declining by 20% by 2009, in tandem with the scaling up of interventions such as provision of antiretroviral therapy (ART) [48], [49]. In Kenya, overall AIDS-related deaths fell 29% between 2002 and 2007 [45]; in the HDSS study area, all-cause and AIDS/TB-associated mortality in adults declined 34% and 26%, respectively, between 2003 and 2008 [14]. During that period, HIV treatment and care centres expanded from 1 to 17, with a corresponding increase in HIV services among HIV-positive HDSS residents [14], with a higher proportion of females to males enrolled in the HIV clinic services. In our current analyses, in the same HDSS study area, all-cause and CD associated mortality among young females halved – suggesting successful targeting of this population group from expanded HIV services. In contrast, the small and non-significant decline in all-cause and CD associated mortality among young men suggest that considerations for mortality are complex, and that young males may not have benefited as much as females from the strengthening of HIV-related services. Earlier infection and treatment seeking among females may have resulted in better identification and access to ART leading to a decrease in mortality. Lessons from interventions which successfully signposted young women into HIV-related services, such as antenatal care and the prevention of mother to child HIV transmission [50]–[52], may also contribute to more successful intervention leading to reduced mortality. For males, programmes need to increase their efforts to identify young HIV-infected men earlier by expanding access to HIV testing, and more importantly to provide them with preventive services (i.e., voluntary medical male circumcision, condoms, STI diagnosis and treatment, etc.) to prevent HIV acquisition. While there was no routine system for generating laboratory-confirmed HIV status among deaths in our population during the study period, current door-to-door HIV testing within the HDSS and future studies on HIV service delivery and HIV associated mortality will allow us to strengthen future analyses and interpretation of the effectiveness of services.

A number of studies have shown mortality reductions among adults in countries of SSA during recent years [10]–[13], but without describing specific reductions among AYA. In Ethiopia, AIDS deaths declined by 21.9 and 9.3% for adult males and females aged 20–64 years, respectively, between 2001 and 2005, and by 38.2 and 42.9% for males and females between 2005 and 2007 [10]. The study explored cause of death recorded in the burial records, a traditional system used in many countries in SSA to register deaths occurring outside of hospital, and calculated a reduction in AIDS deaths in 2007 to be between 56.8% and 63.3%, compared with the expected number in the absence of ART [10]. In Malawi, there was a highly significant linear downward trend in death rates between 2000 and 2007, with a mean annual incremental death rate reduction of 0.52 per 1,000 population per year [12]. Another study, utilising verbal autopsy, demonstrated significant reductions in all-cause and HIV-specific mortality between 2003 and 2009 among South African adults aged 15–49 years but did not disaggregate by age or gender [13].

We have been reticent, in interpreting our data, to place too much credence on cause-specific mortality rates. While an inexact science, verbal autopsy remains one of the only currently available feasible tools to ascribe cause to deaths occurring in community settings in low and middle income countries [53]. Our VA documented that seven in every ten deaths among AYA in the HDSS study site occurred in the home, and of the remainder, the majority dying in hospital were associated with trauma in males. In our study, the majority of deaths were ascribed to communicable diseases, and specifically to HIV. In Ethiopia, VA for diagnosing AIDS mortality had a high sensitivity (0.82) and specificity (0.76) when compared with hospital records reporting HIV serostatus [11]. The model performed better for TB/AIDS combined with a sensitivity and specificity of 0.91 and 0.78, respectively [11]. In Zimbabwe 69% of deaths were accurately ascribed to AIDS/non-AIDS through verbal autopsy [54]. Deaths ascribed to TB and malaria may have been misclassified or have represented co-infection with HIV [28], [55]. Co-morbidity between HIV and TB is well described in the HDSS study area; among a prevalence of 6 per 1,000 pulmonary TB cases in adults of 15 years and older, half tested were found to be HIV positive [29]. Excess mortality in persons 15 years and older who were treated for TB between 2006 and 2008 and HIV infected, was 2-fold higher compared with same aged persons who were HIV infected who did not have TB, when adjusted for age and sex [9].

Understanding the relationship between malaria infection and the 13% of AYA deaths ascribed to this cause is not straightforward. Early adult VA studies had a low sensitivity but high specificity for malaria diagnosis [53], while others excluded malaria in their choice of diseases under examination [56]. A more recent study in Tanzania with comparable endemicity to western Kenya found the sensitivity, specificity, and positive predictive value of malaria diagnosis among older children and adults by VA to be 0.63, 0.90, and 0.70 compared with mortality record death certificates [57]. While malaria is not recognised as a major cause of death among older children in highly endemic malarious areas in SSA [58], a study investigating malaria-associated deaths globally from modelled VA, vital registration, and hospital data, has postulated that malaria deaths among adults may have been under-diagnosed [59]. While malaria-related care for pregnant women [52], [60], and household level protection against transmission [31], has been a focus of attention in the study site, however, we found no significant change over time in mortality rates of malaria-ascribed deaths. Misclassification may also occur with co-infections other than malaria and TB, with duel HIV infection driving mortality [61]. Studies in the area around Lake Victoria have found compelling evidence of co-morbidity with schistosomiasis [36], although schistosomiasis was not recorded or attributed to any CD deaths among AYA during this study period.

NCD mortality primarily represented traumatic injuries for males; and ascription of cause through VA appears less problematical [53]. Absence of diagnostic testing of NCD in this rural setting likely contributed to potential domination of trauma and under-representation of other NCD causes, with attribution to other common causes. While trauma was reported as a cause of death among females, and mortality rates rose relative to other causes, the relatively limited number of deaths ascribed is surprising and not consistent with a national survey documenting that 35.8% of female respondents in western Kenya reported inter-personal violence occurring during the past 12 months [47].

Other NCD causes may be due to a CD; for example, of the CNS deaths 91% were attributed to seizures/epilepsy. Persisting neurological sequelae are recognised to be the long-term consequence of cerebral malaria occurring during childhood [62], [63], thus, a proportion may have malaria as the originating underlying cause in this area which has intense malaria transmission. Neurological complications are also documented in association with opportunistic infections among HIV infected persons despite ART [64]; and HIV was found to be an independent predictor of death among epileptic patients in Ethiopia [65]. Other NCD, such as cardiac, respiratory and renal disease may be falsely attributed to organ failure because of incomplete recall of symptoms remembered by relatives. Excluding deaths from injury and from maternal causes, mortality attributed to NCD contributed a very small proportion of deaths among AYA in rural western Kenya; however, NCD is expected to rise as westernised behaviours and wealth increases, as well as access to diagnostic screening [8], [15], [66]. There remains, nevertheless, a clear need for refinement of VA to further improve sensitivity and specificity of algorithms for both CD and NCD [59], [67]. Evaluation of cause in rural areas, through post-mortem autopsy and biopsies, will contribute towards this and improve the differential diagnoses of deaths. An INDEPTH funded pilot study to evaluate this in our HDSS site is underway.

### Conclusion

This study has identified a halving in the all-cause mortality rate of adolescent and young adult females aged 15–24 years in rural western Kenya over seven years. This runs in tandem with substantive improvements in HIV treatment and care services. While this provides evidence that targeted public health programmes can have significant impact, all-cause and CD mortality rates among females still remain alarmingly high, underscoring the need to further strengthen programmes. The absence of significant reductions among males highlights the need to adapt and strengthen programmes and target strategies to reach both males and females. Expanding and targeting preventive health care services for the main contributors to NCD, such as injuries and maternal health, are called for. Findings highlight the necessity for new research to examine the validity of verbal autopsy for ascribing cause of death in rural SSA.

## References

[1] Patton GC, Coffey C, Sawyer SM, Viner RM, Haller DM, et al (2009) Global patterns of mortality in young people: a systematic analysis of population health data. Lancet 374: 881–892. 19748397 10.1016/S0140-6736(09)60741-8

[2] Viner RM, Coffey C, Mathers C, Bloem P, Costello A, et al (2011) 50-year mortality trends in children and young people: a study of 50 low-income, middle-income, and high-income countries. Lancet 377: 1162–1174. 21450338 10.1016/S0140-6736(11)60106-2

[3] Gore FM, Bloem PJ, Patton GC, Ferguson J, Joseph V, et al (2011) Global burden of disease in young people aged 10–24 years: a systematic analysis. Lancet 377: 2093–2102. 21652063 10.1016/S0140-6736(11)60512-6

[4] Bearinger LH, Sieving RE, Ferguson J, Sharma V (2007) Global perspectives on the sexual and reproductive health of adolescents: patterns, prevention, and potential. Lancet 369: 1220–1231. 17416266 10.1016/S0140-6736(07)60367-5

[5] Amornkul PN, Vandenhoudt H, Nasokho P, Odhiambo F, Mwaengo D, et al (2009) HIV prevalence and associated risk factors among individuals aged 13–34 years in Rural Western Kenya. PLoS One 4: e6470. 19649242 10.1371/journal.pone.0006470 PMC2714463

[6] Blum RW (2007) Youth in sub-Saharan Africa. J Adolesc Health 41: 230–238. 17707292 10.1016/j.jadohealth.2007.04.005

[7] Mathers CD, Loncar D (2006) Projections of global mortality and burden of disease from 2002 to 2030. PLoS Med 3: e442. 17132052 10.1371/journal.pmed.0030442 PMC1664601

[8] Abegunde DO, Mathers CD, Adam T, Ortegon M, Strong K (2007) The burden and costs of chronic diseases in low-income and middle-income countries. Lancet 370: 1929–1938. 18063029 10.1016/S0140-6736(07)61696-1

[9] van’t Hoog A, Williamson J, Sewe M, Mboya P, Odeny L, et al.. (2012) Risk Factors for Excess Mortality and Death in Adults with Tuberculosis in Western Kenya. Int J TB Lung Dis In press. 10.5588/ijtld.12.0135 23131264

[10] Reniers G, Araya T, Davey G, Nagelkerke N, Berhane Y, et al (2009) Steep declines in population-level AIDS mortality following the introduction of antiretroviral therapy in Addis Ababa, Ethiopia. AIDS 23: 511–518. 19169138 10.1097/QAD.0b013e32832403d0 PMC2666986

[11] Tensou B, Araya T, Telake DS, Byass P, Berhane Y, et al (2010) Evaluating the InterVA model for determining AIDS mortality from verbal autopsies in the adult population of Addis Ababa. Trop Med Int Health 15: 547–553. 20214760 10.1111/j.1365-3156.2010.02484.x PMC3901008

[12] Mwagomba B, Zachariah R, Massaquoi M, Misindi D, Manzi M, et al (2010) Mortality reduction associated with HIV/AIDS care and antiretroviral treatment in rural Malawi: evidence from registers, coffin sales and funerals. PLoS One 5: e10452. 20454611 10.1371/journal.pone.0010452 PMC2864258

[13] Herbst AJ, Mafojane T, Newell ML (2011) Verbal autopsy-based cause-specific mortality trends in rural KwaZulu-Natal, South Africa, 2000–2009. Popul Health Metr 9: 47. 21819602 10.1186/1478-7954-9-47 PMC3160940

[14] Gargano JW, Laserson K, Muttai H, Odhiambo F, Orimba V, et al.. (2012) The adult population impact of HIV care and antiretroviral therapy (ART)- nyanza province, Kenya, 2003–2008. AIDS. 10.1097/QAD.0b013e328353b7b9 22441254

[15] Patton GC, Coffey C, Cappa C, Currie D, Riley L, et al (2012) Health of the world's adolescents: a synthesis of internationally comparable data. Lancet 379: 1665–1675. 22538181 10.1016/S0140-6736(12)60203-7

[16] Chandramohan D, Shibuya K, Setel P, Cairncross S, Lopez AD, et al (2008) Should data from demographic surveillance systems be made more widely available to researchers? PLoS Med 5: e57. 18303944 10.1371/journal.pmed.0050057 PMC2253613

[17] Murray CJ, Lopez AD, Feehan DM, Peter ST, Yang G (2007) Validation of the symptom pattern method for analyzing verbal autopsy data. PLoS Med 4: e327. 18031196 10.1371/journal.pmed.0040327 PMC2080648

[18] INDEPTH: Available: http://www.indepth-network.org/. Accessed 2012 Sep 12.

[19] Adazu K, Lindblade KA, Rosen DH, Odhiambo F, Ofware P, et al (2005) Health and demographic surveillance in rural western Kenya: a platform for evaluating interventions to reduce morbidity and mortality from infectious diseases. Am J Trop Med Hyg 73: 1151–1158. 16354829

[20] Odhiambo FO, Laserson KF, Sewe M, Hamel MJ, Feikin DR, et al (2012) Profile: The KEMRI/CDC Health and Demographic Surveillance System–Western Kenya. Int J Epidemiol 41: 977–987. 22933646 10.1093/ije/dys108 PMC12083774

[21] Hamel MJ, Adazu K, Obor D, Sewe M, Vulule J, et al (2011) A reversal in reductions of child mortality in western Kenya, 2003–2009. Am J Trop Med Hyg 85: 597–605. 21976557 10.4269/ajtmh.2011.10-0678 PMC3183762

[22] Phillips-Howard PA, Nahlen BL, Alaii JA, ter Kuile FO, Gimnig JE, et al (2003) The efficacy of permethrin-treated bed nets on child mortality and morbidity in western Kenya I. Development of infrastructure and description of study site. American Journal of Tropical Medicine and Hygiene 68: 3–9. 12749479

[23] Cohen D, Atieno-Odhiambo E (1989) Siaya: The Historical Anthropology of an African Landscape. London: James Currey, Ltd.

[24] Meltzer MI, Terlouw DJ, Kolczak MS, Odhacha A, ter Kuile FO, et al (2003) The household-level economics of using permethrin-treated bed nets to prevent malaria in children less than five years of age. Am J Trop Med Hyg 68: 149–160. 12749499

[25] Cohen MS, Chen YQ, McCauley M, Gamble T, Hosseinipour MC, et al (2011) Prevention of HIV-1 infection with early antiretroviral therapy. N Engl J Med 365: 493–505. 21767103 10.1056/NEJMoa1105243 PMC3200068

[26] Hamel MJ, Greene C, Chiller T, Ouma P, Polyak C, et al (2008) Does cotrimoxazole prophylaxis for the prevention of HIV-associated opportunistic infections select for resistant pathogens in Kenyan adults? Am J Trop Med Hyg 79: 320–330. 18784222

[27] Zeh C, Oyaro B, Vandenhoudt H, Amornkul P, Kasembeli A, et al (2011) Performance of six commercial enzyme immunoassays and two alternative HIV-testing algorithms for the diagnosis of HIV-1 infection in Kisumu, Western Kenya. J Virol Methods 176: 24–31. 21635920 10.1016/j.jviromet.2011.05.021

[28] Van’t Hoog AH, Onyango J, Agaya J, Akeche G, Odero G, et al (2008) Evaluation of TB and HIV services prior to introducing TB-HIV activities in two rural districts in western Kenya. Int J Tuberc Lung Dis 12: 32–38. 18302820

[29] Van’t Hoog AH, Laserson KF, Githui WA, Meme HK, Agaya JA, et al (2011) High prevalence of pulmonary tuberculosis and inadequate case finding in rural Western kenya. Am J Respir Crit Care Med 183: 1245–1253. 21239690 10.1164/rccm.201008-1269OC

[30] Cavanaugh J, Genga K, Marigu I, Laserson K, Ackers M, et al.. (2011) Tuberculosis among Children in Kenya: Epidemiology and Impact of HIV in Two Provinces. J Trop Pediatr. 10.1093/tropej/fmr098 22144009

[31] Hamel MJ, Otieno P, Bayoh N, Kariuki S, Were V, et al (2011) The combination of indoor residual spraying and insecticide-treated nets provides added protection against malaria compared with insecticide-treated nets alone. Am J Trop Med Hyg 85: 1080–1086. 22144448 10.4269/ajtmh.2011.10-0684 PMC3225156

[32] Phillips-Howard PA, Nahlen BL, Kolczak MS, Hightower AW, ter Kuile FO, et al (2003) Efficacy of permethrin-treated bed nets in the prevention of mortality in young children in an area of high perennial malaria transmission in western Kenya. Am J Trop Med Hyg 68: 23–29. 12749482

[33] ter Kuile FO, Terlouw DJ, Phillips-Howard PA, Hawley WA, Friedman JF, et al (2003) Reduction of malaria during pregnancy by permethrin-treated bed nets in an area of intense perennial malaria transmission in western Kenya. Am J Trop Med Hyg 68: 50–60. 12749486

[34] Lindblade KA, Dotson E, Hawley WA, Bayoh N, Williamson J, et al (2005) Evaluation of long-lasting insecticidal nets after 2 years of household use. Trop Med Int Health 10: 1141–1150. 16262739 10.1111/j.1365-3156.2005.01501.x

[35] Karanja DM, Hightower AW, Colley DG, Mwinzi PN, Galil K, et al (2002) Resistance to reinfection with Schistosoma mansoni in occupationally exposed adults and effect of HIV-1 co-infection on susceptibility to schistosomiasis: a longitudinal study. Lancet 360: 592–596. 12241930 10.1016/S0140-6736(02)09781-7

[36] Secor WE, Karanja DM, Colley DG (2004) Interactions between schistosomiasis and human immunodeficiency virus in Western Kenya. Mem Inst Oswaldo Cruz 99: 93–95. 15486642 10.1590/s0074-02762004000900016

[37] Melman SD, Steinauer ML, Cunningham C, Kubatko LS, Mwangi IN, et al (2009) Reduced susceptibility to praziquantel among naturally occurring Kenyan isolates of Schistosoma mansoni. PLoS Negl Trop Dis 3: e504. 19688043 10.1371/journal.pntd.0000504 PMC2721635

[38] Brooks JT, Ochieng JB, Kumar L, Okoth G, Shapiro RL, et al (2006) Surveillance for bacterial diarrhea and antimicrobial resistance in rural western Kenya, 1997–2003. Clin Infect Dis 43: 393–401. 16838225 10.1086/505866

[39] Armah GE, Sow SO, Breiman RF, Dallas MJ, Tapia MD, et al (2010) Efficacy of pentavalent rotavirus vaccine against severe rotavirus gastroenteritis in infants in developing countries in sub-Saharan Africa: a randomised, double-blind, placebo-controlled trial. Lancet 376: 606–614. 20692030 10.1016/S0140-6736(10)60889-6

[40] Dubois AE, Crump JA, Keswick BH, Slutsker L, Quick RE, et al (2010) Determinants of use of household-level water chlorination products in rural Kenya, 2003–2005. Int J Environ Res Public Health 7: 3842–3852. 21139864 10.3390/ijerph7103842 PMC2996196

[41] Olson CK, Blum LS, Patel KN, Oria PA, Feikin DR, et al (2011) Community case management of childhood diarrhea in a setting with declining use of oral rehydration therapy: findings from cross-sectional studies among primary household caregivers, Kenya, 2007. Am J Trop Med Hyg 85: 1134–1140. 22144458 10.4269/ajtmh.2011.11-0178 PMC3225166

[42] van Eijk AM, Adazu K, Ofware P, Vulule J, Hamel M, et al (2008) Causes of deaths using verbal autopsy among adolescents and adults in rural western Kenya. Trop Med Int Health 13: 1314–1324. 18721187 10.1111/j.1365-3156.2008.02136.x

[43] SAVVY Sample Vital Registration with Verbal Autopsy. Available: http://www.cpc.unc.edu/measure/tools/monitoring-evaluation-systems/savvy. Accessed 2012 Sep 12.

[44] McKenzie D (2005) Measuring inequality with asset indicators. Journal of Population Economics 18: 229–260.

[45] Kenya National AIDS/STI Control Programme (NASCOP) (September 2009) 2007 Kenya AIDS Indicator Survey: Final Report. Nairobi, Kenya: NASCOP.

[46] Clark S, Bruce J, Dude A (2006) Protecting young women from HIV/AIDS: the case against child and adolescent marriage. Int Fam Plan Perspect 32: 79–88. 16837388 10.1363/3207906

[47] Kenya National Bureau of Statistics, ICF Macro (2010) *2008–09 Kenya Demographic and Health Survey: Key Findings*. Calverton, Maryland, USA.

[48] UNAIDS (2010) Global report: UNAIDS report on the global AIDS epidemic 2010. Geneva, Switzerland: Joint United Nations Programme.

[49] WHO (2010) Towards universal access: Scaling up priority HIV/AIDS interventions in the health sector. Geneva, Switzerland World Health Organization.

[50] van’t Hoog AH, Mbori-Ngacha DA, Marum LH, Otieno JA, Misore AO, et al (2005) Preventing mother-to-child transmission of HIV in Western Kenya: operational issues. J Acquir Immune Defic Syndr 40: 344–349. 16249710 10.1097/01.qai.0000160712.86580.ff

[51] van Eijk AM, Bles HM, Odhiambo F, Ayisi JG, Blokland IE, et al (2006) Use of antenatal services and delivery care among women in rural western Kenya: a community based survey. Reprod Health 3: 2. 16597344 10.1186/1742-4755-3-2 PMC1459114

[52] Ouma PO, van Eijk AM, Hamel MJ, Sikuku ES, Odhiambo FO, et al (2010) Antenatal and delivery care in rural western Kenya: the effect of training health care workers to provide “focused antenatal care”. Reprod Health 7: 1. 20429906 10.1186/1742-4755-7-1 PMC2867783

[53] Chandramohan D, Maude GH, Rodrigues LC, Hayes RJ (1998) Verbal autopsies for adult deaths: their development and validation in a multicentre study. Trop Med Int Health 3: 436–446. 9657505 10.1046/j.1365-3156.1998.00255.x

[54] Lopman BA, Barnabas RV, Boerma JT, Chawira G, Gaitskell K, et al (2006) Creating and validating an algorithm to measure AIDS mortality in the adult population using verbal autopsy. PLoS Med 3: e312. 16881730 10.1371/journal.pmed.0030312 PMC1526767

[55] van Eijk AM, Ayisi JG, ter Kuile FO, Misore AO, Otieno JA, et al (2003) HIV increases the risk of malaria in women of all gravidities in Kisumu, Kenya. AIDS 17: 595–603. 12598780 10.1097/00002030-200303070-00015

[56] Quigley MA, Chandramohan D, Setel P, Binka F, Rodrigues LC (2000) Validity of data-derived algorithms for ascertaining causes of adult death in two African sites using verbal autopsy. Trop Med Int Health 5: 33–39. 10672203 10.1046/j.1365-3156.2000.00517.x

[57] Setel PW, Whiting DR, Hemed Y, Chandramohan D, Wolfson LJ, et al (2006) Validity of verbal autopsy procedures for determining cause of death in Tanzania. Trop Med Int Health 11: 681–696. 16640621 10.1111/j.1365-3156.2006.01603.x

[58] Reyburn H, Mbatia R, Drakeley C, Bruce J, Carneiro I, et al (2005) Association of transmission intensity and age with clinical manifestations and case fatality of severe Plasmodium falciparum malaria. JAMA 293: 1461–1470. 15784869 10.1001/jama.293.12.1461

[59] Murray CJ, Rosenfeld LC, Lim SS, Andrews KG, Foreman KJ, et al (2012) Global malaria mortality between 1980 and 2010: a systematic analysis. Lancet 379: 413–431. 22305225 10.1016/S0140-6736(12)60034-8

[60] van Eijk AM, Ayisi JG, Slutsker L, Ter Kuile FO, Rosen DH, et al (2007) Effect of haematinic supplementation and malaria prevention on maternal anaemia and malaria in western Kenya. Trop Med Int Health 12: 342–352. 17313505 10.1111/j.1365-3156.2006.01787.x

[61] Abu-Raddad LJ, Patnaik P, Kublin JG (2006) Dual infection with HIV and malaria fuels the spread of both diseases in sub-Saharan Africa. Science 314: 1603–1606. 17158329 10.1126/science.1132338

[62] Carter JA, Mung’ala-Odera V, Neville BG, Murira G, Mturi N, et al (2005) Persistent neurocognitive impairments associated with severe falciparum malaria in Kenyan children. J Neurol Neurosurg Psychiatry 76: 476–481. 15774431 10.1136/jnnp.2004.043893 PMC1739592

[63] Ngoungou EB, Poudiougou B, Dulac O, Dicko A, Boncoeur MP, et al (2007) [Persistent neurological sequelae due to cerebral malaria in a cohort of children from Mali]. Rev Neurol (Paris) 163: 583–588. 17571026 10.1016/s0035-3787(07)90464-6

[64] Oliveira JF, Greco DB, Oliveira GC, Christo PP, Guimaraes MD, et al (2006) Neurological disease in HIV-infected patients in the era of highly active antiretroviral treatment: a Brazilian experience. Rev Soc Bras Med Trop 39: 146–151. 16699639 10.1590/s0037-86822006000200002

[65] Amare A, Zenebe G, Hammack J, Davey G (2008) Status epilepticus: clinical presentation, cause, outcome, and predictors of death in 119 Ethiopian patients. Epilepsia 49: 600–607. 18325017 10.1111/j.1528-1167.2008.01556.x

[66] Mayosi BM, Flisher AJ, Lalloo UG, Sitas F, Tollman SM, et al (2009) The burden of non-communicable diseases in South Africa. Lancet 374: 934–947. 19709736 10.1016/S0140-6736(09)61087-4

[67] Murray CJ, Lopez AD, Shibuya K, Lozano R (2011) Verbal autopsy: advancing science, facilitating application. Popul Health Metr 9: 18. 21794169 10.1186/1478-7954-9-18 PMC3160911

